# Guanine nucleotide exchange factors and colon neoplasia

**DOI:** 10.3389/fcell.2024.1489321

**Published:** 2024-10-18

**Authors:** Lea-Pearl Njei, Natalia Sampaio Moura, Alyssa Schledwitz, Kelly Griffiths, Kunrong Cheng, Jean-Pierre Raufman

**Affiliations:** ^1^ Department of Biological Sciences, University of Maryland, Baltimore County, Baltimore, MD, United States; ^2^ Department of Medicine, Division of Gastroenterology and Hepatology, University of Maryland School of Medicine, Baltimore, MD, United States; ^3^ Department of Biochemistry and Molecular Biology, University of Maryland School of Medicine, Baltimore, MD, United States; ^4^ Biomedical Laboratory Research and Development Service, Veterans Affairs Maryland Healthcare System, Baltimore, MD, United States; ^5^ Marlene and Stewart Greenebaum Comprehensive Cancer Center, University of Maryland Medical Center, Baltimore, MD, United States

**Keywords:** colorectal cancer, guanine nucleotide exchange factors, βPak-interacting exchange factor, GEF-H1, Tiam1, Rho GTPases, muscarinic receptors

## Abstract

Despite many diagnostic and therapeutic advances, colorectal cancer (CRC) remains the second leading cause of cancer death for men and women in the United States. Alarmingly, for reasons currently unknown, the demographics of this disease have shifted towards a younger population. Hence, understanding the molecular mechanisms underlying CRC initiation and progression and leveraging these findings for therapeutic purposes remains a priority. Here, we review critically the evidence that canonical and noncanonical actions of guanine nucleotide exchange factors (GEFs) play important roles in CRC evolution. Rho GEF GTPases, which switch between inactive GDP-bound and active GTP-bound states, are commonly overexpressed and activated in a variety of cancers, including CRC, and may be tractable therapeutic targets. In addition to comprehensively reviewing this field, we focus on Rho/Rac GEFs that are involved in regulating key functions of normal and neoplastic cells like cell polarity, vesicle trafficking, cell cycle regulation, and transcriptional dynamics. Prime examples of such Rho/Rac GEFs include βPak-interacting exchange factor (βPix), a Rho family GEF for Cdc42/Rac1, Tiam1, GEF-H1, RGNEF, and other GEFs implicated in CRC development and progression. Throughout this analysis, we explore how these findings fill key gaps in knowledge regarding the molecular basis of colon carcinogenesis and how they may be leveraged to treat advanced CRC. Lastly, we address potential future directions for research into the role of GEFs as CRC biomarkers and therapeutic targets. In this regard, leveraging the noncanonical actions of GEFs appears to provide a relatively unexplored opportunity requiring further investigation.

## 1 Introduction

Notwithstanding advances in screening and diagnosis, for both men and women colorectal cancer (CRC) remains a common cause of morbidity and mortality; worldwide, in 2022, lung cancer was the most common cause of cancer death (18.7% of total cancer deaths), followed by CRC (9.3%) and breast cancer (6.9%) (Bray et al., 2024). Over the past few decades, high-income countries, predominantly the United States, have experienced increased rates of CRC diagnosis among adults younger than 50 years–although the increased prevalence of obesity, physical inactivity, environmental toxins, and exposure to antibiotics that alter the gut microbiome may play a role in this phenomenon, the precise causes of this worrisome trend remain uncertain (Bray et al., 2024). Moreover, standard chemotherapy and even innovative targeted treatments for cancers that have advanced beyond the colon demonstrate limited efficacy and duration of action, generally extending life by only months (Alese et al., 2023). A commonly used chemotherapy regimen for advanced CRC is FOLFIRI − an acronym for folinic acid (FOL), fluorouracil (F, 5-FU), and the topoisomerase inhibitor irinotecan (IRI). Genotyping tumors can further personalize treatment options by targeting epidermal growth factor receptors (e.g., cetuximab), angiogenesis (e.g., bevacizumab), *KRAS* or *BRAF* mutations (e.g., encorafenib), and immune checkpoint blockade (e.g., pembrolizumab). Unfortunately, only the minority of colon cancers that are mismatch repair-deficient are likely to respond to immune checkpoint blockade, and then only transiently (Alese et al., 2023). Commonly, tumor cell heterogeneity, the development and selection for drug resistance, and other factors soon result in a diminished or lack of response to all current therapeutic approaches.

Consequently, CRC remains an important health concern and active focus of investigation. In the following sections, we review critically the evidence that canonical and noncanonical actions of guanine nucleotide exchange factors (GEFs) play important roles in CRC evolution. Rho GEF GTPases, that facilitate switching between inactive GDP-bound and active GTP-bound states, are commonly overexpressed and activated in CRC (Cao et al., 2019; Wahoski and Singh, 2024). We examine how these observations fill key gaps in knowledge regarding the molecular basis of colon carcinogenesis and, more importantly, how they may be leveraged for therapeutic purposes.

## 2 GEF regulation of normal and cancer cell dynamics

GEFs, and their counterpart GTPase-activating proteins (GAPs), play key roles in catalyzing activation and inactivation of key signal transduction molecules (Table 1). For example, numerous GEFs and GAPs may regulate the activity of the same Rho GTPase (Vigil et al., 2010; Kotelevets and Chastre, 2020). GEFs catalyze and thereby speed the transition between GDP-bound inactive and GTP-bound active molecules. Structurally, most Rho GEFs contain Dbl (DH) and pleckstrin (PH) homology regulatory domains; the requirement of these domains for a particular function of a GEF is commonly used to determine whether the actions are non-canonical or canonical, i.e., requiring functional DH and PH domains (Mosaddeghzadeh and Ahmadian, 2021).

**TABLE 1:** Overview of underlying mechanisms and functional effects of GEFs currently known to be involved in the development and progression of CRC. GEF, guanine nucleotide exchange factor; CRC, colorectal cancer; βpix, βPak-interacting exchange factor; ALDH, aldehyde dehydrogenase; CAF, cancer-associated fibroblast; FOLFOX, folinic acid, fluorouracil, oxaliplatin; FOLFIRI, folinic acid, fluorouracil, irinotecan; EMT, epithelial-to-mesenchymal transition.

GEF	Alternate names	Associated GTPases	Mechanisms in CRC	Functional effects	References
βPix	ARHGEF7, RhoGEF7, Cool-1	Cdc42, Rac1	Overexpression allows βPix to bind to β-catenin and translocate to the nucleus, resulting in transcription of β-catenin target genes	CRC cell proliferation, migration, invasion	Keum et al. (2021); Chahdi and Raufman (2013)
GEF-H1	ARHGEF2	RhoA	Overexpression accelerates cytoskeletal remodeling and cell cycle progression	CRC cell proliferation, invasion, metastasis; shorter overall survival and disease-free survival	Cao et al. (2019); Nie et al. (2009)
Tiam-1	none	Rac1	Overexpression promotes CRC cell stemness via Rac1 activation, overexpression of Nanog, Oct-4, ALDH; Tiam-1 overexpression in CAFs contributes to CRC stemness; Tiam-1 promotes tumor metastasis via the histone methyltransferase NDS2	CRC cell migration, invasion, metastasis; poorer response to FOLFOX and FOLFIRI, shorter overall survival and disease-free survival	Izumi et al. (2019); Song et al. (2023); Kotelevets and Chastre (2020); Zhu et al. (2015)
Rgnef	ARHGEF28, p190RhoGEF	RhoA	Overexpression stimulates FAK-dependent signaling and transcription of genes regulating cell motility and detachment from primary tumors	CRC cell migration, invasion	Kleinschmidt et al. (2019); Yu et al. (2011)
GEFT	ARHGEF25	Cdc42, Rac1, RhoA	Overexpression is associated with microsatellite instability, mismatch repair defects, and promotion of EMT	Shorter overall survival; CRC vessel carcinoma emboli and lymph node metastasis	Wang et al. (2019)
Ephexin4	ARHGEF16	Rac1, RhoG	Overactivation of RhoG/PI3K signaling promotes cell migration and resistance to anoikis	CRC cell proliferation, migration, invasion	Kawai et al. (2013)
GEF5	ARHGEF5	RhoA, RhoB	Overactivation of Rho/ROCK/MLC and PI3K/AKT signaling promotes tumor growth, invasion, resistance to apoptosis, and EMT	CRC cell proliferation, migration, invasion, metastasis; associated with shorter overall survival only in the context of low CDH1 expression and/or SNAI1 overexpression	Komiya et al. (2016)

The canonical actions of βPix, GEF-H1, and Tiam1 include the regulation of actin cytoskeleton dynamics, which is essential for cell movement and stability. These GEFs also influence cell polarity, vesicle trafficking, and cell cycle progression − attributes vital for maintaining the invasive and metastatic capabilities of cancer cells (Varma et al., 2010; Aseervatham, 2020; Cheng et al., 2021; Song et al., 2023). Non-canonical actions involve interactions with other signaling pathways and transcription factors, further enhancing the role of GEFs in CRC pathogenesis.

By regulating the spatial and temporal activation of Rho GTPases and thereby altering cell polarity and vesicle trafficking, GEFs contribute to the invasive and metastatic phenotype of CRC. This is crucial for the dynamic cytoskeletal reorganization and the formation of cellular protrusions that drive cell migration and, for cancer cells, invasion (e.g., membrane ruffles, lamellipodia, filopodia, invadopodia, and microtentacles) (Whipple et al., 2007; Aseervatham, 2020). Rho GTPases play a pivotal role in regulating cellular apoptosis, proliferation, survival, adhesion, and membrane trafficking.

## 3 Key GEFs in CRC progression

**FIGURE 1:**
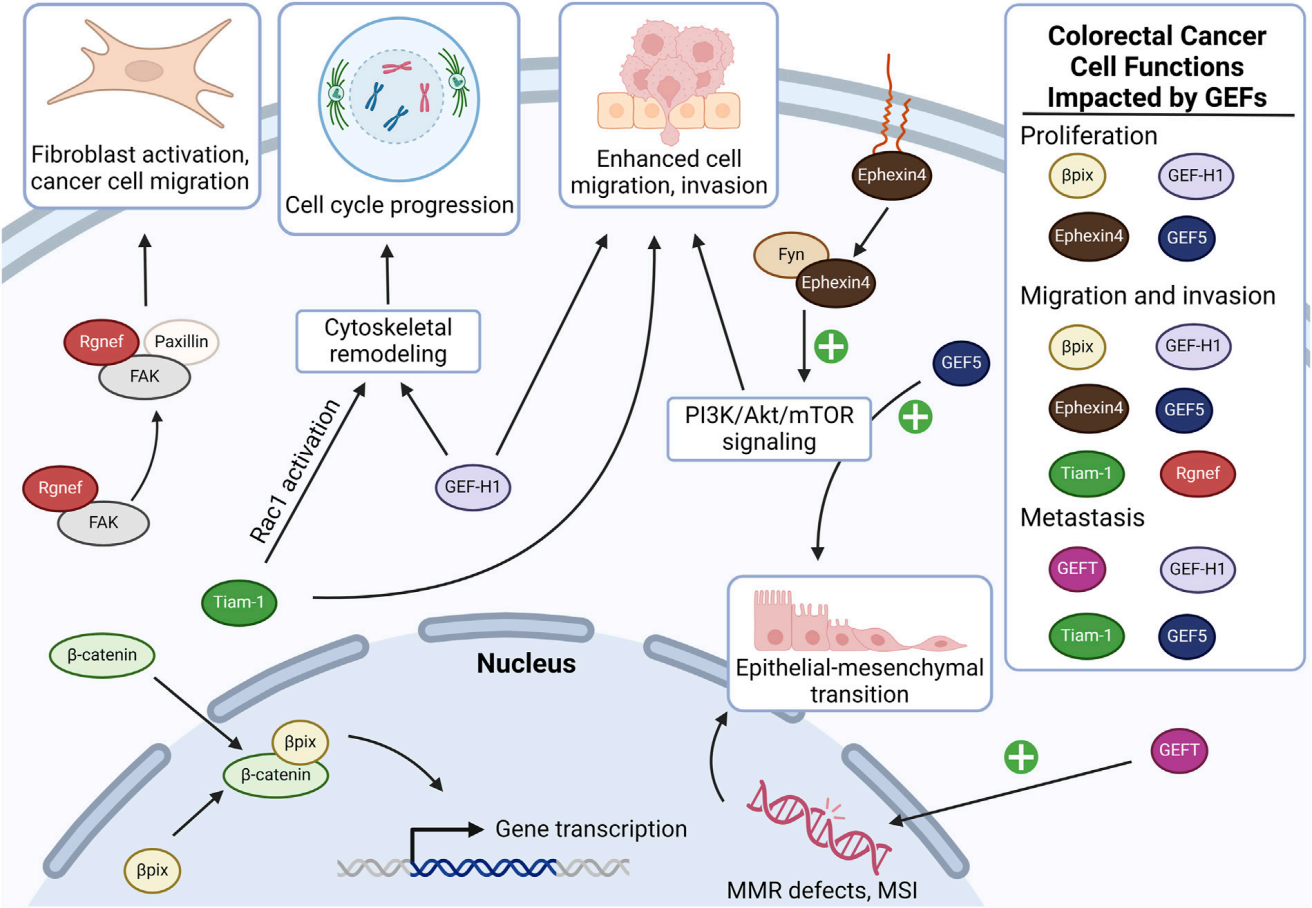
Overview of the actions of GEFs implicated in colon cancer carcinogenesis and progression. In colon cancer cells, Rgnef interacts with FAK and paxillin, promoting activation of focal adhesions in cancer-associated fibroblasts, which enhances cancer cell motility and invasiveness. βPix is overexpressed in colon cancer and stabilizes β-catenin, promoting the transcription of genes involved in cancer proliferation and invasion (see additional details in Figure 2). Tiam-1 contributes to colon cancer cell migration, invasion, and metastasis by promoting cell cycle progression, as well as by promoting Rac1-dependent oncogene transcription. Similarly, GEF-H1 overexpression promotes colon cancer cell cycle progression via RhoA. When stabilized by Fyn, Ephexin4 upregulates phosphatidylinositol 3-kinase (PI3K) signaling which contributes to cell migration and proliferation at the invasive front. GEF5 also activates PI3K signaling, resulting in upregulation of epithelial-mesenchymal transition in cancer cells. GEFT overexpression also upregulates colon cancer cell epithelial-to-mesenchymal transition, albeit by impairing the cell’s ability to mitigate DNA damage. GEF, guanine nucleotide exchange factor; FAK, focal adhesion kinase; MMR, mismatch repair; MSI, microsatellite instability. Created in BioRender.

A pattern of complex molecular changes resulting from the combination of genomic instability, environmental influences, and other factors underlies the development and progression of CRC. With progressive chromosomal instability, KRAS mutations constitutively activate downstream RAS signaling largely through the actions of GEF-catalyzed turnover of GDP (inactive) to GTP (active). In cancer as in non-neoplastic cells, GEFs play a critical role in activating Rho GTPases by promoting the exchange of GDP for GTP, transitioning GTPases from the dormant to active state. GTPase activation regulates signal transduction pathways controlling cytoskeletal arrangement, cell proliferation, and cell invasion–all fundamental attributes of cancer cells (Cao et al., 2019; Mosaddeghzadeh and Ahmadian, 2021). The RAS superfamily comprises a conserved family of Rho GTPases (Pino and Chung, 2010), including RhoA, Rac1, and Cdc42 (Haga and Ridley, 2016). As summarized in Table 1, work from several laboratories demonstrated that altered Rho GTPase signaling correlates with the progression of colon neoplasia. RhoA activation leads to the formation of basal stress fibers essential for cytoskeleton-driven cell motility, whereas Rac1 and Cdc42 activation results in the formation of lamellipodia and filopodia, respectively (Leve and Morgado-Diaz, 2012). Here, we focus only on GEFs implicated in CRC development and progression, highlighting actions that hasten epithelial-to-mesenchymal transition (EMT), invasion into neighboring tissues, and ultimately the spread of cancer cells to other organs. Prominent GEFs that activate Rho GTPases and promote CRC progression include βPix, GEF-H1, Tiam-1, Rgnef, GEFT, Ephexin4, and GEF5 (Yu et al., 2011). Figure 1 provides an overview of their actions.

βPak-interacting exchange factor (βPix; aka ARHGEF7), a Cdc42/Rac1GEF, interacts and forms complexes with a variety of proteins, thereby playing a pivotal role in actin-mediated processes such as cell migration (Keum et al., 2021). βPix is overexpressed in human colon cancer cells and modulates CRC progression in part by virtue of its ability to alter β-catenin signaling and the transcriptional activity of β-catenin target genes (Figure 1) (Chahdi and Raufman, 2013). By a noncanonical mechanism independent of its GEF activity, βPix binds to β-catenin via its DH and c-terminal leucine zipper dimerization domains (Chahdi and Raufman, 2013; Cheng et al., 2021). βPix is essential for normal intestinal development (Cheng et al., 2021). Thus, in addition to associating with nuclear β-catenin, βPix regulates fundamental cellular dynamics often hijacked by cancer cells. Conditional knockout of βPix from normal murine intestinal epithelial cells impairs enterocyte proliferation, villus maturation, mucosal defense, maintenance of intestinal permeability, resistance to toxic injury, and overall intestinal homeostasis (Cheng et al., 2021). On a molecular level, βPix deletion dysregulates ROCK/myosin light chain 2 signaling (Cheng et al., 2021), a mechanism that normally controls actomyosin dynamics, focal adhesions, cell cycle control, gene transcription, and vesicular trafficking (Jin and Blikslager, 2020).

Guanine Nucleotide Exchange Factor-H1 (GEF-H1; aka ARHGEF2), another Rho GEF, regulates cell cycle and transcriptional dynamics and modulates CRC progression via activation of RhoA and cell cycle regulation (Figure 1). GEF-H1 is associated with microtubule networks and its activity is regulated by their polymerization state. Upon microtubule depolymerization, GEF-H1 is released and activates RhoA and Erk/Ras/MAPK signaling independently of its GEF properties (Varma et al., 2010; Cullis et al., 2014; Cao et al., 2019). This signaling cascade stimulates actomyosin contractility, focal adhesion formation, and advances the leading edges of invading cells. Notably, treatment with microtubule disrupting agents commonly used against cancer such as vinca alkaloids and taxanes may unintentionally release GEF-H1, thereby activating its downstream effectors. In addition to modulating cytoskeletal remodeling and cell polarity, GEF-H1 activation also promotes cell cycle progression via indirect upregulation of cyclin-D1 and other cell cycle controls (Nie et al., 2009). GEF-H1 overexpression in CRC is associated with increased cell proliferation and metastases, supporting its important role in cancer progression (Cao et al., 2019).

T-lymphoma invasion and metastasis-inducing protein 1 (Tiam1) activates Rac1, thereby promoting cell migration and invasion by stabilizing junctional complexes and enhancing cell polarity (Figure 1). Tiam1 overexpression in CRC is associated with a poor prognosis and reduced sensitivity to chemotherapy (i.e., reduced overall and progression-free survivals) (Izumi et al., 2019). Tiam1-mediated Rac1 activation triggers several downstream pathways, including those involved in cytoskeletal reorganization and cell adhesion (Minard et al., 2005; Song et al., 2023). Downstream of EGFR/PI3K signaling, AKT1 phosphorylates Tiam1 leading to Rac1 activation, stabilization of Rac-1 by 14-3-3, and subsequent expression of oncogenes such as c-myc and cyclin-D1 (Zhu et al., 2015). Tiam1 directly regulates high mobility group box 1 protein (HMGB1), an autophagy-associated protein passively secreted in response to cell stress that may contribute to CRC chemoresistance (Liu et al., 2008; Chen et al., 2022). Tiam1 overexpression in CRC cells increases their migratory and invasive capabilities. For example, SW480 human colorectal carcinoma cell lines with enhanced migratory potential exhibit four- to five-fold increased Tiam1 protein and mRNA expression. Unlike parental cells, when these migratory sublines were implanted into the cecum of athymic mice, they formed tumors in the spleen, liver, and lungs, strongly supporting a role for Tiam1 in CRC dissemination (Kotelevets and Chastre, 2020). Ectopic expression of Tiam1 in SW480 cells induced morphological changes and increased migration, underscoring Tiam1’s role in promoting the metastatic phenotype of CRC cells through Rac1 activation and subsequent cytoskeletal reorganization and enhancement of cell adhesion (Kotelevets and Chastre, 2020; Song et al., 2023). These observations highlight the role of Tiam1 in promoting cell migration and invasion, and colon cancer cell dissemination.

Rgnef, (aka ARHGEF28 and p190RhoGEF), impacts cancer progression by regulating RhoA activity, an essential feature of focal adhesion dynamics and cell motility (Kleinschmidt et al., 2019). Rgnef’s unique ability to bind focal adhesion kinase (FAK) via its C-terminus–a feature not shared with other GEFs–has important implications for cancer progression via FAK/RhoA/paxillin signaling (Figure 1) (Miller et al., 2012). This interaction is crucial for the proper localization and activation of FAK at focal adhesions. In normal intestinal cells, binding of the gastrointestinal hormone gastrin to the CCK2 receptor allows the Gα_13_ subunit of the heterotrimeric G protein to interact with Rgnef at the cell membrane, thereby activating FAK by Y397 phosphorylation which in turn induces paxillin activation and focal adhesion formation on fibroblasts (Masia-Balague et al., 2015). Paxillin, a focal adhesion adaptor protein, plays a key role in cell motility and migration (Lopez-Colome et al., 2017). Upon integrin binding to extracellular matrix proteins like fibronectin, FAK is phosphorylated at tyrosine residue 397 (Y397), triggering downstream signaling cascades essential for tumor cell migration and invasion. Rgnef enhances FAK localization to early peripheral adhesions and promotes its activation upon fibronectin binding. This scaffolding role of Rgnef is dependent on its PH domain but independent of GEF activity. PH domain mutations inhibit adhesion formation, FAK localization, and Y397 phosphorylation without disrupting Rgnef-FAK interaction, indicating the PH domain’s crucial role in Rgnef’s scaffolding function. Interestingly, a GEF-inactive Rgnef mutant remains capable of rescuing FAK-Y397 phosphorylation and early adhesion localization but not paxillin-Y118 phosphorylation, thereby suggesting Rgnef GEF activity is specifically required for key downstream signaling events (Miller et al., 2013). The Rgnef-FAK axis impacts CRC progression by enhancing cell motility, regulating adhesion dynamics, and interacting with the tumor microenvironment. By modulating focal adhesion turnover, Rgnef-FAK signaling promotes detachment of cancer cells from primary tumors and their invasive capacity and alters the tumor microenvironment in ways that promote tumor growth and metastasis (Yu et al., 2011). The Rgnef/FAK complex also enhances colon cancer cell motility by translocating to premature focal adhesion sites thereby stimulating the formation of invadopodia (Yu et al., 2011).

Widely expressed, GEFT (aka ARHGEF25) determines whether mesenchymal progenitor cells become adipogenic or myogenic. This property of GEFT is hijacked by CRC cells to stimulate EMT (Figure 1). Thus, in CRC, increased GEFT expression is associated with an increased risk of metastasis and reduced overall survival (Wang et al., 2019).

Downstream of the Ephrin family of receptor tyrosine kinases, Ephexin4 (aka ARHGEF16), is implicated in CRC cell growth, proliferation, invasion, migration, and resistance to cell detachment-induced anoikis (Kawai et al., 2013). In non-neoplastic cells, Ephexin4 helps remove apoptotic cells by activating Rac1 downstream of the RhoG-Elmo1-Dock4 pathway (Lee et al., 2014). In CRC, independent of this pathway, after interacting with phosphorylated (S897) ephrin type-A receptor 2, Ephexin4 activates RhoG/PI3K signaling thereby enhancing cell migration and resistance to anoikis (Figure 1) (Kawai et al., 2013). Ephexin4-induced migration and invasion requires synonymous expression of Fyn, a Src family nonreceptor tyrosine kinase that stabilizes and prevents Ephexin4 degradation (Yu et al., 2020); notably, this requirement provides a potential therapeutic target.

Upregulated GEF5 (aka ARHGEF5) promotes EMT and plays a critical role in podosome and invadopodia formation. GEF5 is phosphorylated by Src and Fyn tyrosine kinases leading to dual activation of the Rho/ROCK/MLC and PI3K/AKT pathways. In HCT116 human colon cancers cells, GEF5 activity stimulates cytoskeletal actin rearrangement. Together, these signaling events increase tumor growth, cell motility, and resistance to apoptosis. This phenotype appears confined to mesenchymal-like cells, indicating that GEF5’s tumorigenic activity likely depends on EMT status, thereby providing a potential therapeutic target (Figure 1) (Komiya et al., 2016).

## 4 Insights into the regulation of noncanonical actions of βPix in CRC

In contrast to their canonical actions, little is known regarding the upstream regulation of the noncanonical actions of GEFs in CRC. Several insights derived from studies focused on elucidating the mechanisms whereby the activation of cholinergic muscarinic receptors modulates colon cancer development and progression. Of the five muscarinic receptor subtypes, designated M_1_R-M_5_R and encoded, respectively, by *CHRM1*-*CHRM5*, M_3_R/*CHRM3* expression is increased in CRC, whereas M_1_R/*CHRM1* expression is reduced (Cheng et al., 2017; Alizadeh et al., 2022). Blocking M_3_R expression or activation inhibits human colon cancer cell proliferation; mice lacking M_3_R expression have substantially fewer colon tumors than littermate control animals (Cheng et al., 2014).

In the course of these studies, our laboratory made the serendipitous observation that activating M_3_R upregulated βPix/β-catenin colocalization in both the cytoplasm and nucleus of colon cancer cells, providing a potential mechanism whereby M_3_R modulates the expression of β-catenin target genes, including the genes encoding cMyc (*MYC*), cyclin D1 (*CCND1*), and cyclooxygenase-2 (*PTGS2*) (Cheng et al., 2023). We found that M_3_R protein and mRNA (*CHRM3*) overexpression in CRC correlated with nuclear and cytoplasmic colocalization of βPix and β-catenin. Moreover, M_3_R activation with muscarinic receptor agonists, namely acetylcholine and carbamylcholine, stimulated nuclear colocalization of βPix and β-catenin–actions mimicked by epidermal growth factor receptor (EGFR) and protein kinase-C (PKC) activation (Cheng et al., 2023). EGFR and PKC-α were previously identified as downstream effectors of M_3_R activation (Cheng et al., 2003; Said et al., 2017). Supporting their role in post-M_3_R signal transduction, blocking either EGFR or PKC activation with chemical inhibitors effectively attenuated acetylcholine-induced nuclear colocalization of βPix with β-catenin (Cheng et al., 2023).

**FIGURE 2:**
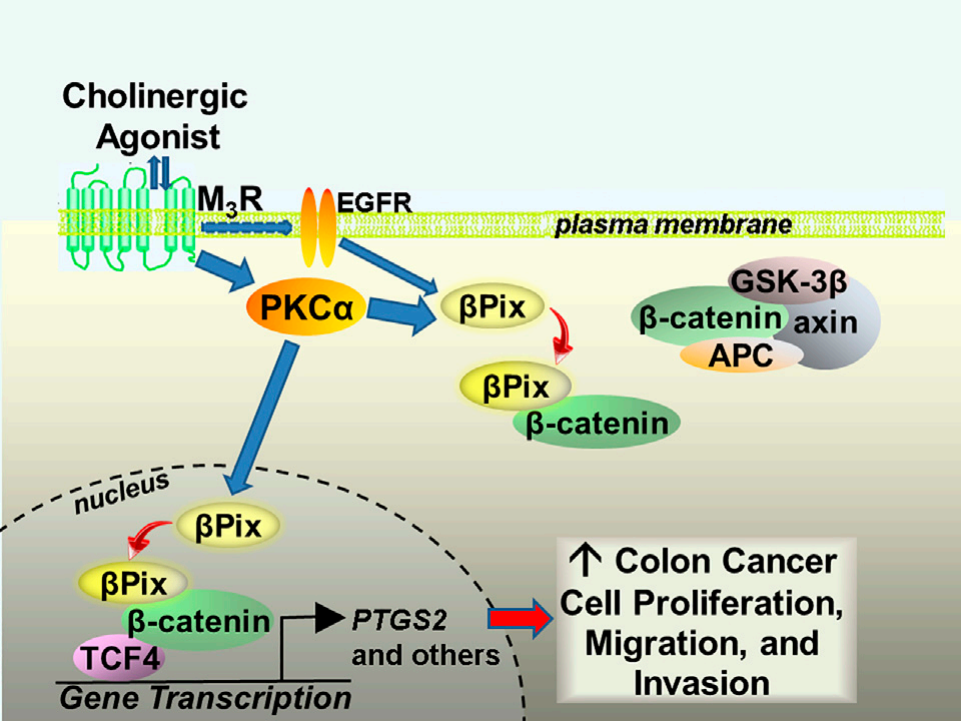
Proposed mechanism whereby M3 muscarinic receptor activation stimulates noncanonical binding of βPix to β-catenin in the cytoplasm and nucleus of human colon cancer cells. M_3_R activation results in transactivation of EGFR and activation of PKCα. *In vitro* findings suggest that βPix binding to β-catenin in the nucleus enhances β-catenin binding to the key transcription factor TCF4. This action induces the expression of many β-catenin target genes, including *PTGS2*, that promote colon cancer cell proliferation, migration, and invasion. M_3_R, M3 muscarinic receptors; EGFR, epidermal growth factor receptors; PKCα, protein kinase C-alpha; GSK-3β, glycogen synthase kinase-3 beta; APC, adenomatous polyposis coli; TCF4, transcription factor-4; *PTGS2*, prostaglandin endoperoxide synthase 2 (cyclooxygenase 2).

Based on these and other experimental findings (Cheng et al., 2023), it was proposed that M_3_R activation stimulates βPix/β-catenin complex formation in both the cancer cell cytoplasm and nucleus (Figure 2). Our preliminary findings (Cheng et al., 2023) suggest that the association of βPix with β-catenin in the nucleus enhances binding of β-catenin to transcription factor-4 (*TCF4*), a key transcription factor that when bound by β-catenin induces the expression of a multitude of β-catenin target genes including *MYC*, *CCND1* and *PTGS2* (Lecarpentier et al., 2019), illustrated in Figure 1. Additional findings supporting this mechanism included reduced colon tumor formation in muscarinic agonist-treated mice lacking intestinal epithelial cell βPix expression and reduced *MYC*, *CCND1* and *PTGS2* gene and protein expression in colon adenocarcinomas resected from those mice (Cheng et al., 2023).

Similar enhancement of β-catenin target gene expression was reported as a consequence of Tiam1 association with the β-catenin/TCF transcription factor complex (Buongiorno et al., 2008); increased expression of Tiam1 was previously shown to stimulate colon cancer cell invasion (Liu et al., 2005), migration, and tumor formation and metastases in CRC animal models (Minard et al., 2005; Malliri et al., 2006). Although it is unknown whether these effects are also regulated by muscarinic receptor activation, these observations suggest that GEF association with the β-catenin/TCF transcription factor complex may be a common mechanism downstream of canonical Wnt/β-catenin signaling as well as mediating the noncanonical actions of GEFs. As Tiam1 and βPix are β-catenin target genes, this may also provide positive feedback loops for these multi-functional GEFs (Buongiorno et al., 2008).

## 5 Conclusion and future directions

As described above, GEFs are potential biomarkers for early detection of neoplastic transformation and molecular targets for therapeutic intervention. In this review, we identified gaps in knowledge that provide grist for further investigation. Future research foci may include studies to determine whether the activation of muscarinic or other receptors implicated in CRC progression regulates other GEFs besides βPix, e.g., Tiam1 association with the β-catenin/TCF transcription factor complex. GEF-H1/RhoA/MLC2 signaling is reported to play a role in colon cancer invasion and metastasis (Cao et al., 2019), but the mechanisms are imprecisely understood and the upstream regulation of GEF-H1 is uncertain–the same is true regarding a limited understanding of how upstream signaling may modulate the actions in CRC of GEFT, Ephexin4, and GEF5. Additionally, knowledge gaps remain regarding the mechanisms whereby GEFs promote CRC, e.g., identifying binding partners necessary for GEF activation, and determining their precise role in governing neoplastic attributes including invasion, migration, and metastasis as well as resistance to apoptosis and anoikis. For example, Tiam1 interacts with protease activated receptor 1 (Par1) at the leading edge of invasive breast cancer cells (Adams et al., 2010), but whether this occurs in CRC is unexplored. Investigating whether GEF-induced effects depend on CRC cell EMT status, e.g., as noted with GEF5, is also worth exploring. Finally, little is currently known regarding potential roles in CRC progression for novel GEFs, e.g., ARHGEF12 and ARHGEF33, whose overexpression in CRC is associated with poor outcomes (Wu et al., 2023).

In summary, abundant evidence supports the concept that canonical and noncanonical actions of GEFs play a key role in CRC pathogenesis and progression. We believe that targeting these actions has great therapeutic potential. Lastly, we apologize to investigators for any work we unintentionally neglected and to readers for the relatively brief summaries of GEF actions necessitated by space limitations. We encourage interested parties to pursue the references cited.
